# The underlying molecular mechanisms and biomarkers between periodontitis and COVID-19

**DOI:** 10.1186/s12903-023-03150-4

**Published:** 2023-07-26

**Authors:** Danlei Qin, Feiyan Yu, Dongchao Wu, Chong Han, Xuemin Yao, Lulu Yang, Xi Yang, Qianqian Wang, Dongning He, Bin Zhao

**Affiliations:** 1 Shanxi Province Key Laboratory of Oral Diseases Prevention and New Materials, Shanxi Medical University School and Hospital of Stomatology, No 63, New South Road, Yingze District, Taiyuan, 030001 Shanxi China; 2 Department of Medical Imaging, Shanxi Medical University, Taiyuan, 030001 Shanxi China

**Keywords:** COVID-19, Periodontitis, Transcription factors, Plasma cells, Susceptibility

## Abstract

**Objective:**

Emerging evidence shows the clinical consequences of patient with COVID-19 and periodontitis are not promising, and periodontitis is a risk factor. Periodontitis and COVID-19 probably have a relationship. Hence, this study aimed to identify the common molecular mechanism that may help to devise potential therapeutic strategies in the future.

**Material and methods:**

We analyzed two RNA-seq datasets for differential expressed genes, enrichment of biological processes, transcription factors (TFs) and deconvolution-based immune cell types in periodontitis, COVID-19 and healthy controls. Relationships between TFs and mRNA were established by Pearson correlation analysis, and the common TFs-mRNA regulatory network and nine co-upregulated TFs of the two diseases was obtained. The RT-PCR detected the TFs.

**Results:**

A total of 1616 and 10201 differentially expressed gene (DEGs) from periodontitis and COVID-19 are found. Moreover, nine shared TFs and common biological processes associated with lymphocyte activation involved in immune response were identified across periodontitis and COVID-19. The cell type enrichment revealed elevated plasma cells among two diseases. The RT-PCR further confirmed the nine TFs up-regulation in periodontitis.

**Conclusion:**

The pathogenesis of periodontitis and COVID-19 is closely related to the expression of TFs and lymphocyte activation, which can provide potential targets for treatment.

**Supplementary Information:**

The online version contains supplementary material available at 10.1186/s12903-023-03150-4.

## Clinical relevance

SARS-CoV-2, an important cause of coronavirus disease in 2019 (COVID-19), has become a great threat to mankind all over the world. COVID-19 can exacerbate the development of systemic diseases in humans, including hypertension, diabetes, cardiovascular disease, older age and obesity. The link between COVID-19 and chronic diseases has attracted extensive attention and research, and periodontitis is no exception. However, no one understands what is the exact biological mechanism of action between COVID-19 and human periodontitis. Thus, it is crucial to identify potential risk factors for COVID-19 in periodontitis. In this study, we found that the pathogenesis of periodontitis and COVID-19 is closely related to the expression of transcription factors (TFs) and lymphocyte activation, which can provide potential targets for treatment. Furthermore, our findings emphasize the importance of early treatment periodontitis during pandemics to reduce the host's susceptibility to COVID-19.

## Introduction

Coronavirus disease in 2019 (COVID-19) is associated with SARS-CoV-2, and has become a great threat to mankind all over the world [1, 2]. COVID-19 can exacerbate the development of systemic diseases in humans, including hypertension, diabetes, cardiovascular disease, older age and obesity [3–6]. The link between COVID-19 and chronic diseases has attracted extensive attention and research, and periodontitis is no exception [7]. However, no one understands what is the exact biological mechanism of action between COVID-19 and human periodontitis. Thus, the identification of potential risk factors is of importance for COVID-19 patients to developing efficient individualized and population-based preventive strategies.

Periodontitis is one of the most common non-communicable chronic inflammatory diseases. It is estimated that severe periodontitis accounts for 9% of the general population and is the sixth largest epidemic disease in the world [8], which is characterized by inflammation caused by periodontal plaque microorganisms and loss of periodontal attachment [9]. As an initiator of inflammation, plaque biofilm can trigger systemic immune and inflammatory responses in the host [10, 11]. Given the impact of periodontitis on the systemic conditions, it is reasonable to assume that there is a possible linkage between periodontitis and COVID-19. Some unspecific oral lesions have been associated with COVID-19. These include dry mouth, oral vesiculobullous or pustulous lesions, lip necrosis, fissured or depapillated tongue, or erythematous or hemorrhagic mucosal lesions [7]. Recent case–control studies have identified periodontitis as a factor leading to COVID-19 complications (such as death, ICU admission and need for assisted ventilation) and elevated levels of blood markers, and COVID-19 patients are more likely to suffer from gingival bleeding and plaque accumulation [12, 13]. Another study also showed that the risk of death from COVID-19 is higher for patients with painful/bleeding gums [14]. A meta-analyses of epidemiological studies indicated that periodontitis subjects are more likely to experience a more severe course of COVID-19. Periodontitis was associated with fourfold increased odds of hospitalisation, sixfold of requiring assisted ventilation, and more than sevenfold of death due to COVID-19 complications [15]. Moreover, SARS-CoV-2 is transmitted and pathogenic through the oral mucosa, as the main host cell receptor for COVID-19, angiotensin-converting enzyme II (ACE2) plays a crucial role in the final infection by virus entering cells [16, 17], and there is also a high expression of ACE2 in the oral mucosa (primarily in tongue epithelial cells) [18]. These studies indicate that the oral cavity may be a potentially high-risk route of SARS-CoV-2 infection. Therefore, periodontal care and oral hygiene monitoring could contribute to preventing coronavirus infection during and after the pandemic of COVID-19. Meanwhile, periodontitis increases the burden of systemic inflammatory and the level of inflammatory mediators. At the same time, the cytokine storm in severe COVID-19 infection is similar [19]. A study suggested that high TGF-β1 production may be a protective factor for periodontitis [20]. However, studies have shown that TGF- β Pathway is involved in the development of alveolar fibrosis in the late stage of COVID-19 [21]. Circulating Endothelial progenitor cells (EPCs) level and function may serve as both biomarkers of vascular function. Periodontitis has been associated with endothelial dysfunction, and with lower lower EPCs (CD133 + /KDR +) levels [22]. Activation of cytokine-mediated inflammation, endothelial dysfunction and thrombus formation and this way vascular cardiopulmonary collapse leads to poor prognostic result of the COVID-19 process. Based on this, it can be inferred that periodontitis may be an indirect risk factor of COVID-19 [23]. Aside from the inflammatory response, immune response also participates in the development of diseases. Plasma cells have attracted much attention because they help the host immune system to eliminate pathogens as soon as possible, single cell sequencing showed two diseases can cause increased levels of plasma cells [24, 25], which further indicates that the molecular regulation of the two diseases may be correlated. A variety of regulators control the increased levels of inflammatory factors and plasma cells. As the key regulatory factors for genetic information transfer from DNA to messenger RNA, transcription factors (TFs) play a crucial role in governing both the temporal and spatial expression of genes [26]. TFs (IRF4 and XBP1) are involved in the synthesis of proteins in plasma cells [27, 28]. Indeed, TFs can also regulate a variety of mRNAs and construct gene regulatory networks of diseases. Consequently, we believe that it is essential to identify common TFs and ultimate synergistic biomolecular pathways induced by COVID-19 and periodontitis, which may provide a potential therapeutic target for patients with COVID-19 periodontitis. Despite the fact that both COVID-19 and periodontitis have many common complications, the common molecular alterations between COVID-19 and periodontitis have not yet been investigated.

**Fig. 1:**
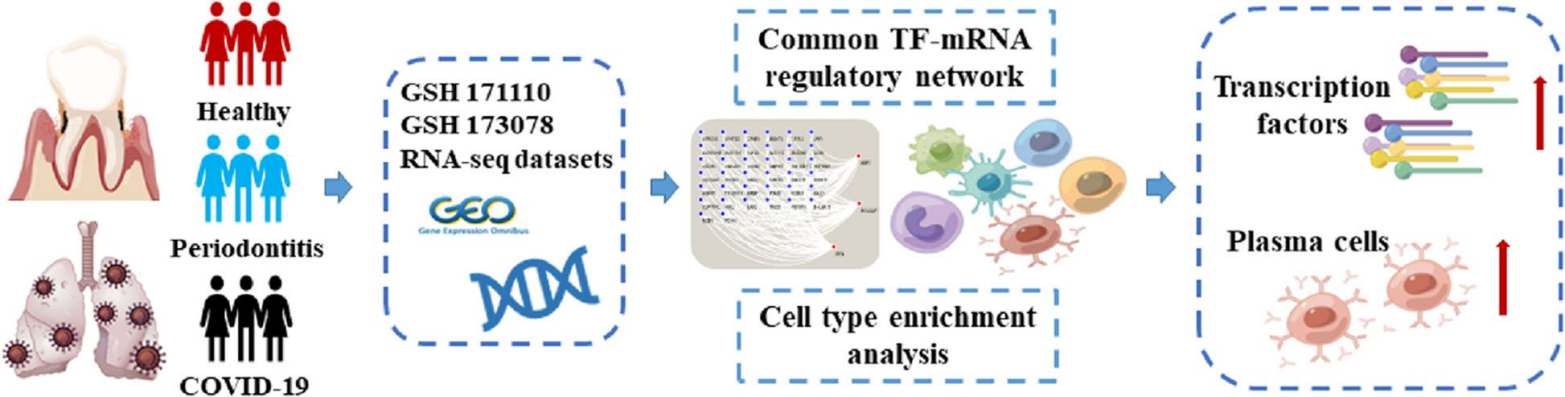
Schematic illustration of the overall general workflow of this study

In this study, we explored the possible molecular mechanisms linking the two diseases to provide the basis and direction for future research through bioinformatics approaches. The Gene Expression Omnibus (GEO) database was used to clarify the biological relationship among periodontitis and COVID-19. Initially, differentially expressed gene (DEGs) and TF-mRNA regulatory pairs were identified for the two diseases. Then, co-upregulated TFs among the two diseases were clarified, and the expression of TFs in periodontal tissue was verified by RT-PCR. Here, functional enrichment analysis of TFs was further carried out to understand the biological processes of these two diseases. Finally, a cellular convolution analysis was performed to identify the immune cell subtypes of the two diseases.The workflow of our study is shown in Fig. 1.

## Materials and methods

### Acquisition of the datasets

To determine shared genetic interrelations among COVID-19 and periodontitis, we assumed RNA-seq datasets from the GEO database of the National Center for Biotechnology Information (NCBI) (https://www.ncbi.nlm.nih.gov/geo/). The periodontitis dataset was (GEO accession ID: GSE173078) human periodontal tissue, containing 12 periodontal samples and 12 healthy controls, which was sequenced by Illumina HiSeq 4000 platform (Homo sapiens). The GEO accession ID of the COVID-19 dataset is GSE171110, which is transcriptome analysis of peripheral sera from patients with COVID-19 throughout the sequencing Illumina HiSeq 2500 platform (Homo sapiens) for extracting RNA sequence. This dataset included peripheral blood mononuclear cells (PBMCs) samples from 44 COVID-19 subjects and 10 healthy controls. And we integrated batches of genomic data to eliminate the unwanted variation in data caused by differences in technical factors across batches.

### Data processing and DEGs identification

To determine the DEG, several statistical operations were performed on the datasets.. The limma (Linear Models for Microarray Analysis) R package has been used to perform statistical tests to identify DEGs. In addition, the Benjamini–Hochberg false discovery rate method is used to provide a good balance between the discovery of statistically significant genes and the limitation of false positives. In this work, cut-off criteria (*P*-value < 0.05 and |logFC|≥ 1.0) was used to detect significant DEGs in two datasets.

### Gene Ontology (GO) and Kyoto Encyclopedia of Genes and Genomes (KEGG) functional enrichment analysis

Gene set enrichment analysis is an important analytical work, which aims to reveal the biological significance of the identified DEGs. The enrichment analysis was performed using the widely utilized tool Metascape. GO analysis reflects the biological process of genes. The KEGG pathway is known to master the pathways and metabolic processes. In this study, we used GO and KEGG enrichment to determine the shared pathways among COVID-19 and periodontitis. Metascape identifies the most significant term in each cluster, and describing the cluster in bar graph. The *P* value < 0.05 was considered as a standard metric for quantifying the top listed pathways.

### Recognition of TFs and mRNAs engage with DEGs

TFs are proteins that attach to specific genes and control the transcription rate of genetic information. So this is very important for molecular research.. By Metascape, the TFs regulating the expression of the DEGs was predicted using the TRRUST database, which is an artificially curated human and mouse transcriptional regulatory network database. We identified the TFs of the two diseases through the TRRUST database. We screened out TF-mRNA interaction pairs with strong correlation in two diseases through Spearman correlation analysis, respectively. The filter condition is *P* < 0.05 & correlation coefficient > 0.7. Later, we searched for the common TF-mRNA interaction pairs and co-upregulated TFs among COVID-19 and periodontitis. The GO and KEGG enrichment analysis of the co-upregulated TFs was performed by Metascape.

### xCell deconvolution analysis

The xCell abundance scores of 64 cell types were evaluated by deconvolution integration gene enrichment analysis, including multiple adaptive and innate immune cells, epithelial cells and extracellular matrix cells, including 48 cells closely related to the tumor microenvironment. Different immune cell subtypes can be used as an index to evaluate the prognosis of the disease. In this study, we use the xCell to analyze the different immune cell subtypes between periodontitis and COVID-19.

### Quantitative realtime-PCR validation

To verify the expression of immune-related TFs in periodontitis lesions. Six gingival tissues with periodontitis lesions from periodontitis patients and six healthy gingival tissues from tooth extraction patients were analyzed. All participants received informed consent. The study was approved by Shanxi Provincial Stomatological Hospital Institutional Committee.Total RNAs of the above samples were extracted by TRIzol reagent (Thermo Fisher Scientific). Extracted RNAs were reverse transcribed into complementary DNA (cDNA) using the EasyScript One-Step gDNA Removal and cDNA Synthesis SuperMix (Transgen Biotech, Beijing, China). Real-time PCR was performed on a CFX96 Touch Deep Well Real-Time PCR Detection System (Bio-Rad, USA) according to the manufacturer’s instructions for the iTaq Universal SYBR Green Supermix (Bio-Rad, USA).β-actin was used as an internal reference. Relative expression level was computed using 2^−ΔΔCt^ method.

## Results

### Identification DEGs of periodontitis and functional enrichment analysis

**Fig. 2:**
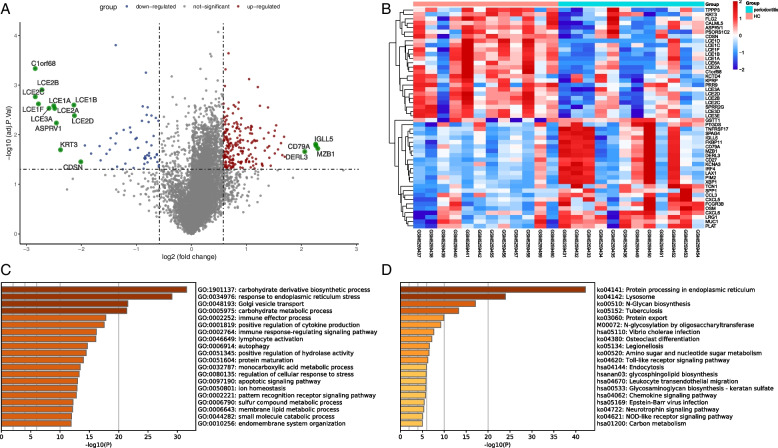
Comparison of DEGs expression data between periodontitis and healthy individuals, and DEGs functional enrichment analysis. **A** Red dots and blue dots presented in volcano plots represent the significant DEGs of periodontitis, red dots represented up-regulated mRNA, blue dots represented down-regulated mRNA, in which some representative genes were highlighted with green dots. The criteria chosen to be considered as DEGs are (i) absolute value of log2 fold-change>1 (ii) adjusted *P*-value < 0.05. **B** Heatmap illustrating the top DEGs across periodontitis and healthy control; **C** Biological process-related GO terms identification results according to combined score. The higher the enrichment score, the higher number of genes are involved in a certain ontology; **D** Pathway analysis of DEGs between periodontitis and healthy control through KEGG

To study the relationships and implications of periodontitis and COVID-19, we first analyzed the GSE173078 dataset of periodontitis patients and identified 1616 genes (including 1464 up-regulated mRNA and 152 down-regulated mRNA) that were differentially expressed compared with healthy control (Supplementary table 1). The first 10 up-regulated mRNAs and down-regulated mRNAs are displayed in Table 1. Volcanic map and heatmap illustrates the DEGs among periodontitis and healthy control (Fig. 2A, B). We performed functional enrichment using the Metascape tool to identify the signaling pathways and functional GO terms (biological processes) in DEGs. GO analysis indicated that immune effector process, immune response-regulating signaling pathway and lymphocyte activation are among the top GO terms for periodontitis (Fig. 2C). Osteoclast differentiation, toll-like receptor signaling pathway and chemokine signaling pathway are involved in the occurrence and progression of periodontitis (Fig. 2D). Among them, toll-like receptors could regulate the innate immune response of the host to periodontal bacteria, and can be used as a bridge between innate immune response and adaptive immune response. Chemokines are a family of cytokines, which have chemotaxis in essence and lead to the recruitment of inflammatory cells [19].

**Table 1:**
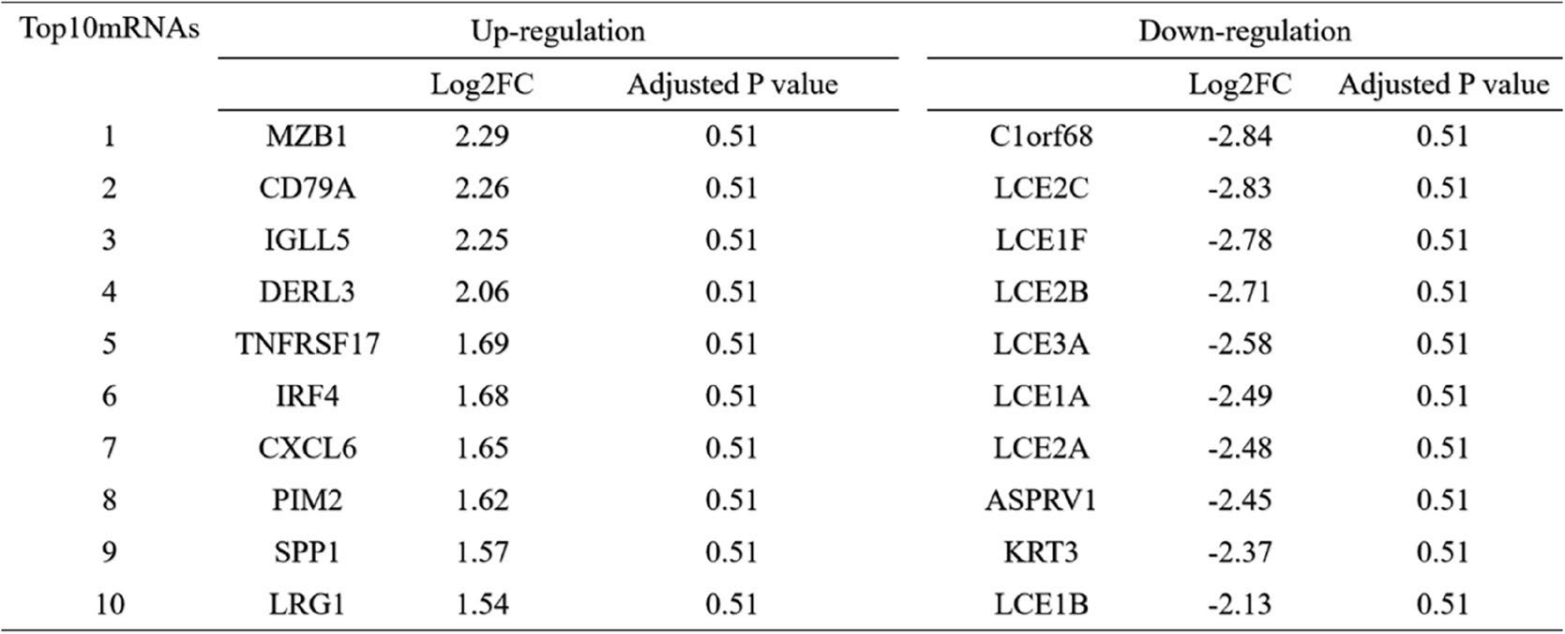
The top 10 up-regulated and down-regulated mRNAs in the DEGs of the periodontitis and healthy control

### Identification DEGs of COVID-19 and functional enrichment analysis

**Fig. 3:**
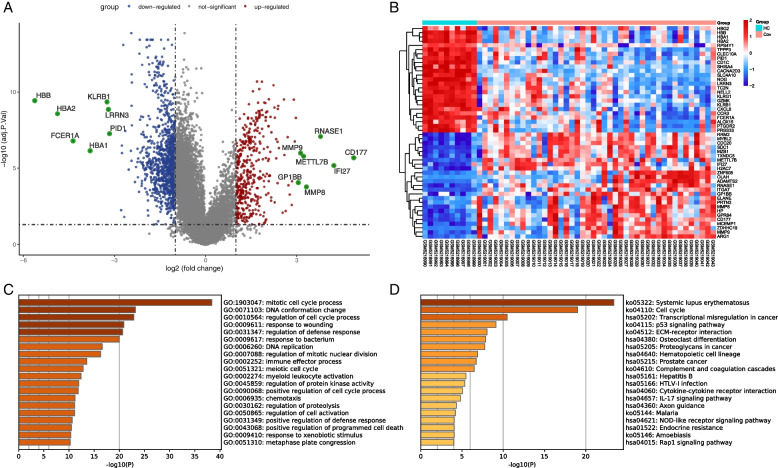
Comparison of DEGs expression data between COVID-19 and healthy individuals, and DEGs functional enrichment analysis. **A** Red dots and blue dots presented in volcano plots represent the significant DEGs of COVID-19, red dots represented up-regulated mRNA, blue dots represented down-regulated mRNA, in which some representative genes were highlighted with green dots. The criteria chosen to be considered as DEGs are (i) absolute value of log2 fold-change>1 (ii) adjusted *P*-value < 0.05. **B** Heatmap illustrating the top DEGs across COVID-19 and healthy control; **C** The bar graphs of ontological analysis of DEGs, for the biological process, immune effector process is among the top GO terms; **D** The bar graphs of KEGG pathway enrichment analysis of DEGs among COVID-19 and healthy control

Similarly, 10,201 DEGs were differentially expressed in COVID-19 and healthy controls, including 1993 up-regulated mRNA and 8208 down-regulated mRNA (Supplementary table 2). Then, the top 10 up-regulated mRNAs and down-regulated mRNAs are displayed in Table 2. Compared with healthy controls, the DEGs of COVID-19 are shown in the volcano plot and heatmap (Fig. 3A, B). In a complex disease, multiple signaling pathways and GO terms are involved in disease coordination and progression. A comprehensive gene set enrichment tool Metascape was used to describe the biological process and signal pathway of DEGs. The GO terms were identified as immune effector process, myeloid leukocyte activation and response to xenobiotic stimulus in Fig. 3C. As shown in Fig. 3D, the cytokine—cytokine receptor interaction pathway and IL-17 signaling pathway are common enrichment pathways in COVID-19 and are involved in inflammatory cytokine storms. In addition, We also found some pathways that are associated with liver disease and cancer, such as hepatitis B and prostate cancer. Levels of clotting cascade biomarkers are important in COVID-19 responses and have been used as prognostic biomarkers to predict mortality in patients with COVID-19 [29]. Finally, we found that there is a common GO term between COVID-19 and periodontitis, that is, both diseases are related to the immune effector process.

**Table 2:**
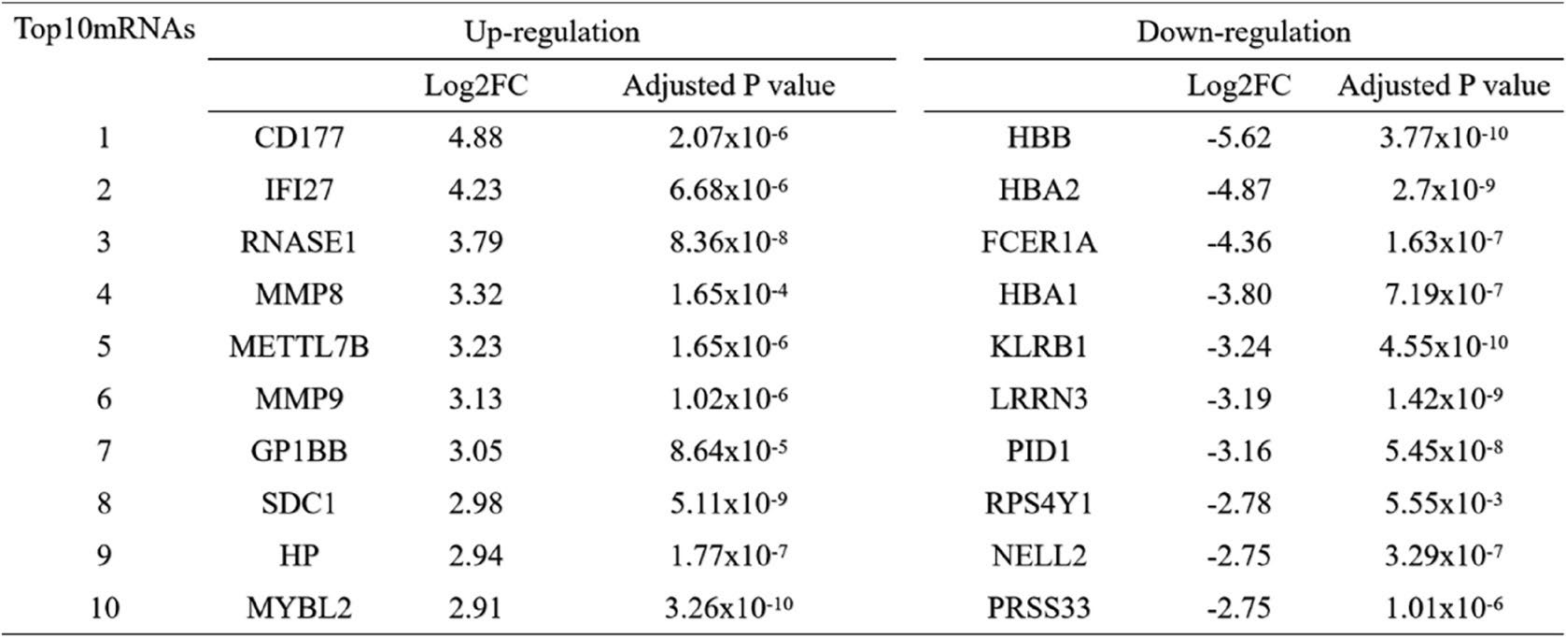
The top 10 up-regulated and down-regulated mRNAs in the DEGs of the COVID-19 and healthy control

### Identification TF-mRNA regulatory network

**Fig. 4:**
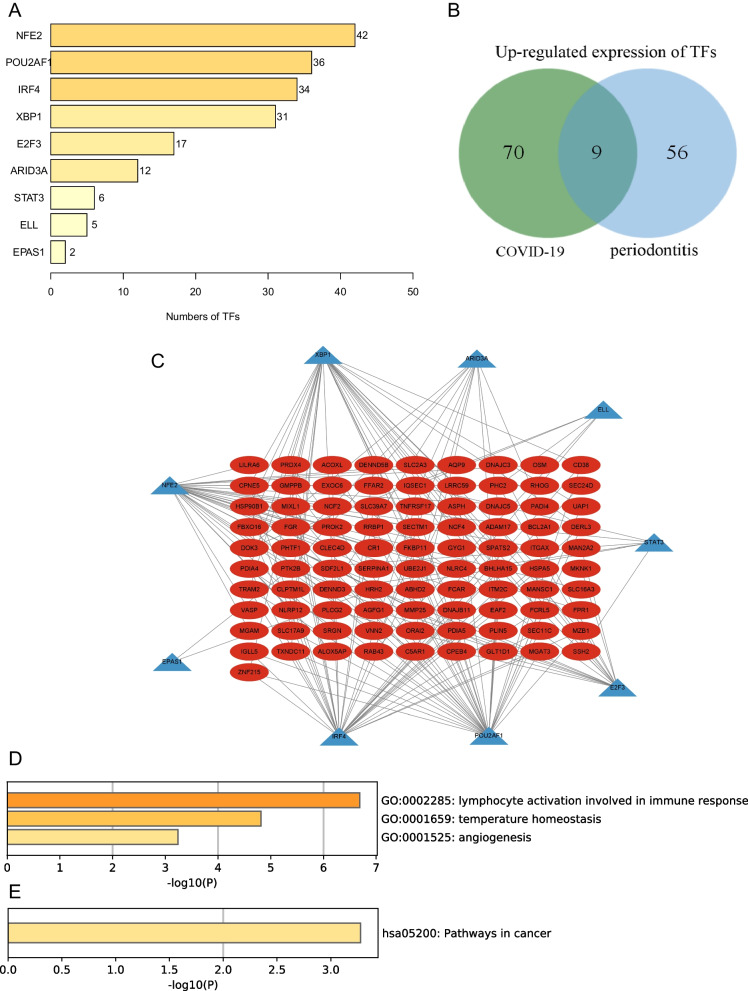
**A** Enrichment of regulator shows NFE2 as the top-ranking TF; **B** Venn diagram showing 9 common up-regulated TFs across COVID-19 and periodontitis; **C** TFs-mRNA core network consists of the 175 pairs of mRNA and TFs among periodontitis and COVID-19, red circles indicate TFs, blue triangle indicate mRNAs, the edges between them mean that the mRNAs were potentially regulated by the TFs; TFs enrichment had been performed by Metascape tool. **D** Go enrichment showed that TFs was mainly involved in lymphocyte activation; (E) KEGG enrichment showed that TFs was involved in cancer related pathways

TF plays an extremely important role in cell proliferation, tissue differentiation and individual development. And it is an important regulatory unit of gene temporal and spatial expression, which can bind to specific DNA motifs and play a role in gene transcription regulation. Thus, firstly, we used TRRUST tool to identify the TFs in the DEGs of the two diseases, we identified 72 TF and 1544 mRNA in the periodontitis DEGs. In the same way, we found that 425 TF and 9776 mRNA for COVID-19 DEGs. Through the correlation analysis of TF and mRNA expression levels among two diseases, we regard the Pearson correlation coefficient of TF-mRNA greater than 0.7 and P value less than 0.05 as a correlation pair. We have obtained 18927 TF-mRNA interaction pair in periodontitis and 769519 TF-mRNA interaction pair in COVID-19. In order to further clarify the common TF-mRNA regulatory network between the two diseases, we found 1335 shared TF-mRNA interaction pairs. Among 1335 TF-mRNA relationship pairs, 175 pairs of TFs were co-upregulated (Supplementary table 3) (including 1 pair of negative regulation, 174 pairs of positive regulation). Ultimately, nine co-upregulated TFs are involved, which are namely NFE2, POU2AF1, IRF4, XBP1, E2F3, ARID3A, STAT3, ELL and EPAS1 (Fig. 4A,B). It is found that NEF2 occupies a core position in the occurrence process of the two diseases. The common TF-mRNA interactions network is displayed in Fig. 4C.

### Nine co-upregulated TFs functional enrichment analysis

GO (biological processes) and KEGG enrichment analysis were used to further studied the common pathways of the co-upregulated TFs. The GO term significantly enriched by the nine co-upregulated TFs between COVID-19 and periodontitis was related to lymphocyte activation involved in immune response (Fig. 4D). As an external stimulus, the SARS-CoV-2 virus will trigger the host immune response, in which lymphocytes will participate [25]. KEGG pathway enrichment analysis showed that these TFs were meaningfully enriched in the development of cancer (Fig. 4E). Some studies have pointed out that cancer with poor immune function is also a susceptibility factor for COVID-19.Cancer patients already exhibit severely weakened and altered immune systems due to the specific cancer therapies, leading to an increased risk of COVID-19. A study observed that COVID-19 patients with cancer showed a higher risk and frequency of severe event occurrences compared with the patients without cancer [30]. The Chinese Centre for Disease Control and Prevention has reported that 5.6% of case fatality rate amongst COVID-19 patients was those with cancer [31].

### Validation of sequencing data using real-time PCR

**Fig. 5:**
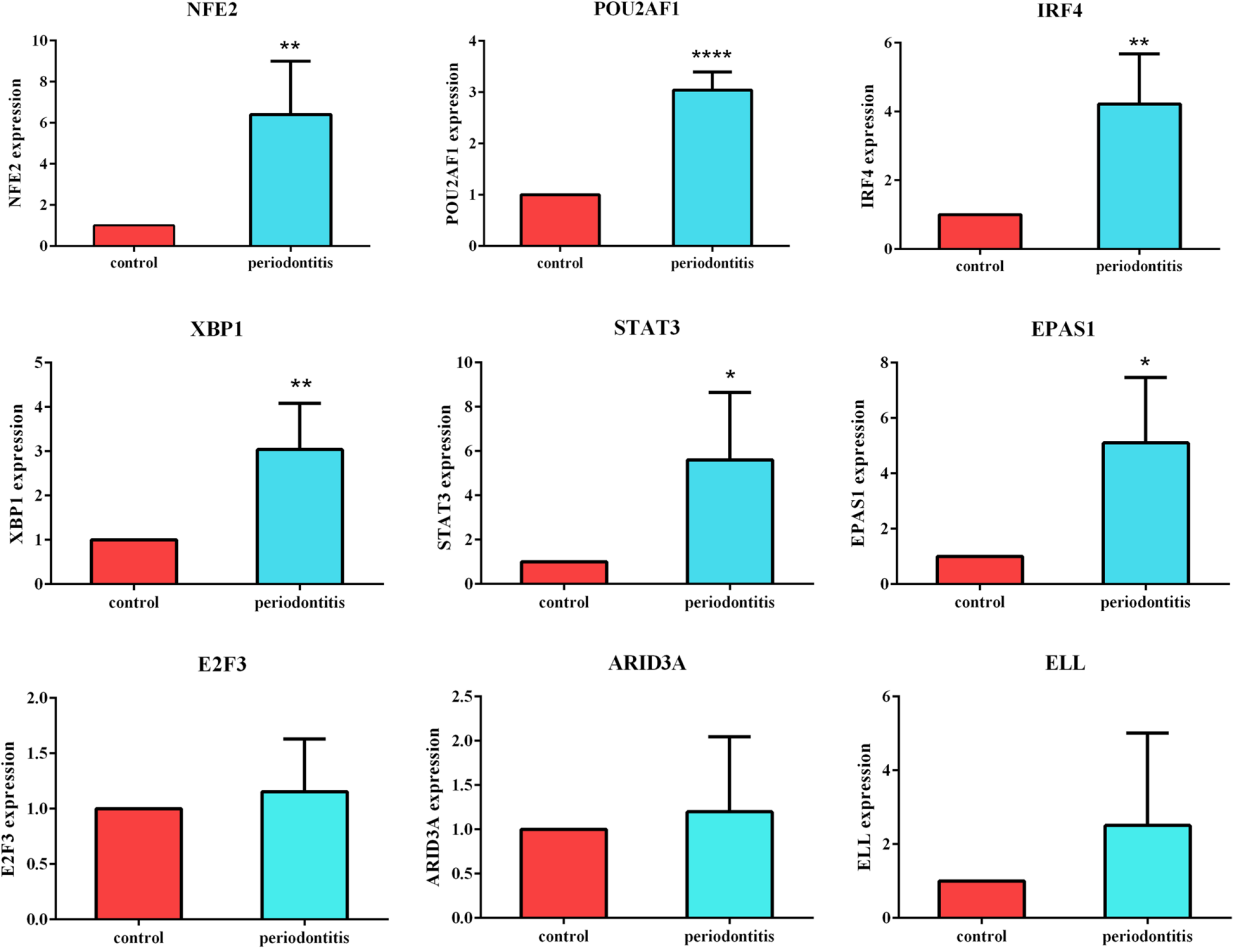
The expression levels of nine co-upregulated TFs(NFE2, POU2AF1, IRF4,XBP1, STAT3, EPAS1, E2F3, ARID3A and ELL) were examined by quantitative real-time polymerase chain reaction. The control represents healthy group. (*p < 0.05, ** *p* < 0.01, ***p < 0.001, ****p < 0.0001)

PCR results were consistent with data analysis, indicating that all TFs in the periodontitis group were up-regulated compared with in the healthy control group.. Our validation results show that there is significant difference in NFE2, POU2AF1, XBP1, IRF4, STAT3, EPAS1 between healthy group and periodontitis group (Fig. 5), however, there is no significant difference in E2F3, ARID3A and ELL among the two groups.

### Plasma cell infiltration between two diseases

**Fig. 6:**
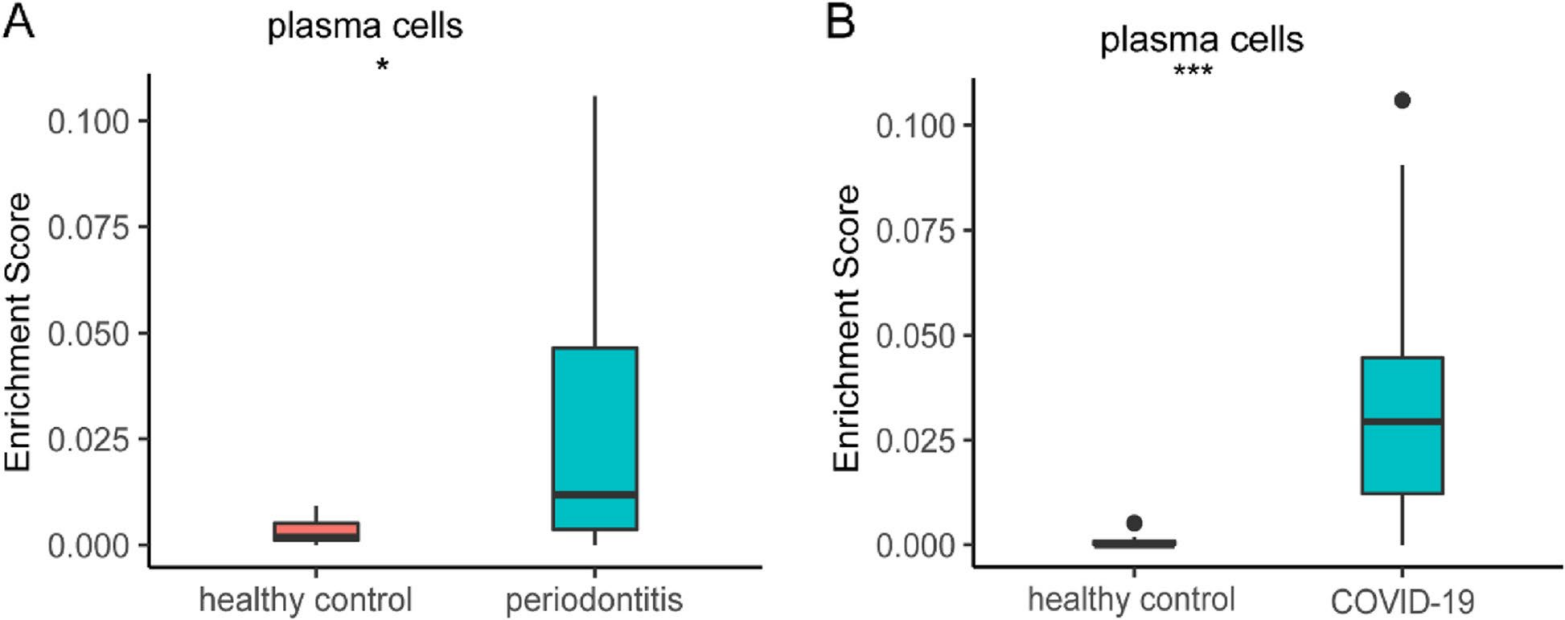
Immune cell composition generated by xCell-inferred enrichment score of cell types across periodontitis, COVID-19 and healthy controls. Compared with healthy controls, plasma cell levels were elevated in periodontitis and COVID-19 (*p < 0.05; **p < 0.01; ***p < 0.001)

Through the previous series of analysis, we concluded that both diseases can cause lymphocyte activation. In order to further understand which lymphocytes are involved in the occurrence and progress of the disease. We performed xCell convolution analysis to further determine immune cell subtypes (Supplementary Fig. 1 and Fig. 2). The xCell results demonstrated that plasma cell was positively enriched in COVID-19 and periodontitis versus healthy individuals (*p* < 0.001) (Fig. 6). We think plasma cells subtypes could play a central role in regulating the host immune response. However, further research is needed to understand the role of other subtypes of immune cells in regulating the development and progression of both diseases.

## Discussion

Complications are associated to a higher risk of developing severe forms of COVID-19, requiring consequent mechanical ventilation and leading to increased mortality. Periodontitis has become a potential risk factor among the complications affecting the prognosis of COVID-19 patients [12, 14]. This may be partly related to the binding of SARS-CoV-2 to ACE2, which is expressed in oral tissues, especially in the epithelial cells of the tongue, bucal mucosa and gingiva [18]. Furthermore, periodontal pathogens promote the increased expression of ACE2 in the oral tissues and may increase the infection rate of SARS-CoV-2 [32]. Interestingly, cytokine storm and expression profile in severe COVID-19 infection are similar to that in periodontitis, suggesting a possible link between periodontitis and COVID-19 and its related complications [19]. Even though several pertinent studies on the connection between COVID-19 and periodontitis have been done, more research are needed to be done on the probable mechanisms and biomarkers that connect the two diseases.

Here, based on the GEO database, we studied gene expression patterns in two RNA-seq datasets from patients with periodontitis and COVID-19, and established a potential molecular association between the two diseases through systematic bioinformatics analysis. We found that TFs and immune response-related pathways play an important role in the pathogenesis of these two disease, which may provide new therapeutic targets for patients with co-morbidity COVID-19 and periodontitis. The nine co-upregulated TFs in both diseases were found by analyzing the datasets. NFE2(nuclear factor erythroid 2) is a key TF regulating antioxidant expression [33]. NFE2 plays a critical role in protecting the host from periodontitis tissue damage. It can up-regulate NFE2-associated antioxidant and detoxification enzymes to enhance the protective effect on cells, and finally decreased inflammatory signal transduction and oxidative damage in tissues [33, 34]. Similarly, in clinical trial, NFE2 activator reduced lung alveolar cells damage in COVID-19 positive patients [35]. Accordingly, we believe that NFE2 activation may be a feasible adjuvant treatment for preventing periodontitis and COVID-19. With regard to POU2AF, researchers observed expression of POU domain class 2–associating factor 1 (POU2AF1) in a genome-wide RNA-seq analysis of human airway epithelium gene expression [36]. And it was identified as a novel host defense regulator in the human airway epithelium [37]. This gene was previously thought to be specifically expressed in lymphocytes. POU2AF1 encodes OCA-B protein(coactivator of OCT2, as a B cell specific TF), plays a pivotal role in the regulation of normal and neoplastic germinal center B cells [38]. Thus, we think that POU2AF1 might be therapeutic targets for COVID-19-related airway diseases. XBP1, as a crucial TF, plays a key role in the endoplasmic reticulum (ER) stress response [39]. Studies have shown that XBP1 regulates the transcription of many genes related lipid (hepatic lipogenesis and adipocyte differentiation) [40], glucose metabolism [41] and immune responses. It also participates in the development and differentiation of various immune cells [42]. XBP1 has been proved to be the necessary TF for mature B lymphocytes to eventually differentiate into plasma cells [27]. Therefore, XBP1 can be an important target for studying various diseases. As a member of the interferon regulatory family of TFs, IRF4, like XBP1, also participates in immune response, cell development and differentiation, growth regulation and metabolism [43]. For example, IRF4 can regulate the development of germinal center B cells and plasma cells [28], and IRF4 is necessary for receptor editing in immunoglobulin gene rearrangement, which is important stage for B cell self-tolerance [43]. Although the specific role of IRF4 in the pathogenesis of periodontitis is poorly defined, however, our analysis found that TRF4 was co-upregulated in both periodontitis and COVID-19, and was involved in the immune response process of the two diseases. Moreover, IRF4 participates in autoimmune diseases such as systemic lupus erythematosus and rheumatoid arthritis [44]. STAT3 is a critical pathway for regulating the immune and inflammatory responses, and it also has important roles in cell proliferation, survival and apoptosis [45]. A recent study indicated that activated STAT3 signaling pathway may contribute to neuroinflammation and cognitive impairment in ligature-induced periodontitis rats [46]. For COVID-19, due to the occurrence of cytokine storm, the over-activation of STAT-3 can play a critical role in the COVID-19 pathogenesis. The IL-6/JAK/STAT-3 axis potently potentiates inflammatory responses [47] and may cause the decrease of lymphocytes in COVID-19 [48]. Thus STAT-3 may be considered as a possible therapeutic target for severe COVID-19. EPAS1, located on chromosome 2, is transcribed into two protein-coding transcripts whose expresion is enhanced in the lungs. EPAS1, also known as Hypoxia inducible factor 2 alpha (HIF2α), play an important role in the transcription of many hypoxia-responsive genes [49]. In addition, EPAS1 has associated with various diseases, such as non-small cell lung cancer [50], paraganglioma and pheochromocytoma [51], and chronic mountain sickness, which can regulate proliferation of erythroblasts [52]. In general, consistent with previous findings, these TFs play an extremely important role in immune inflammatory response. In addition, we discovered TFs, E2F3, ELL and ARID3A are up-regulated in periodontitis tissues compared with the healthy control, however, there was no statistical significance, there may be shortcomings of small sample size. These three TFs have not been reported in other studies and need to be further explored in the future.

Our results also show that lymphocyte activation in immune response is a common biological process of periodontitis and COVID-19. That is, the immune system constantly patrols and monitors the gum environment to regulate local immunity and maintain tissue homeostasis as bacteria invade the connective tissue of the gum. Exactly, which lymphocytes are involved in the development of the disease, we concluded that both diseases cause increased levels of plasma cells through analysis. Some studies have also reported the same results. A high-dimensional single-cell analysis showed that CD4^+^ T cell depletion, T cell differentiation, plasma cell amplification, and the reduced antigen presentation capacity of innate immunity in COVID-19 [20]. In all immune cell clusters, the percentage of plasma cells increased significantly among all five COVID-19 patients compared with healthy controls, which in line with previous research results [53]. A study showed that the risk of death in severe COVID-19 patients with plasma cells detected in peripheral blood was reduced, indicating that plasma cells may play a critical role in the immune response to COVID-19 [54]. Antibody mediated immune response produced by plasma cells plays an important role in SARS-COV-2 infection. The main target of SARS-COV-2 neutralizing antibody is S protein, which binds to the receptor binding domain (RBD) to block the binding of virus with ACE2. The timing of antibodies is regular at different times of viral infection, with one study showing that positive IgA diagnoses are highest (88.2%) in 4–10 days after symptoms appear, which proved that IgA had a good diagnostic effect in the early stage [55]. For periodontitis, previous data analysis also showed plasma cells was elevated in periodontitis tissues [56]. Plasma cells in periodontal tissue mainly secreted IgA and IgG specific for periodontal pathogens to protect the tissue from damage [57]. Moreover, plasma cells potentially participated in and regulated the bone loss through IL-35 and IL-37 [24]. Some cytokines are involved in plasma cell proliferation, differentiation and survival. Apart from cytokine, some TFs, such as PRDM1, XBP1 and IRF4, can regulate plasma cell properties and functions [58, 59]. Our analysis also showed that XBP1 and IRF4 are up-regulated in COVID-19 patients.

While the data supporting an association between periodontitis and COVID-19 is unclear, there is a potential biological link between the two diseases in terms of immunity and inflammation perspective. Based on our findings, we believe TFs can be used as potential therapeutic targets, and immunotherapy and can make major breakthroughs in the future for COVID-19 and periodontitis. Previous studies have found that plasma cells in periodontitis and COVID-19 play an immunomodulatory role by producing IgA [55, 57]. SARS-COV-2 invades human body mainly through respiratory tract, oral mucosa and conjunctival epithelium [18], so it is speculated that mucosal IgA has a protective effect on these physical barriers to a certain extent. Studies have shown that oral and nasal administration of mucosal vaccine targeting SARS-COV-2 RBD can induce the secretion of IgA in mucosa, which could prevent the development of COVID-19 [60, 61]. Mucosal vaccine maybe provide new insights for the treatment of periodontitis and COVID-19 In addition, it is worth noting that COVID-19 will increase the formation of venous thrombosis, and oral anticoagulants can prevent the occurrence of adverse events. For COVID-19 patients who undergo oral anticoagulant, it is necessary to carefully evaluate their conditions during periodontal surgery [62]. The sufficient repair of the endothelial lining of blood vessels with Endothelial progenitor cells (EPCs) treatment may have a crucial role to overcome the vascular collapse driving forces in COVID-19 patients, as well as to modulate human immune system [23].

## Conclusion

In this study, transcriptome analysis was performed to detect common pathways and molecular biomarkers in periodontitis and COVID-19 to help understand the association among these two disease. We performed DEG and pathway analyses between the two datasets and established a common TF mRNA regulatory network and identified the shared up-regulated TFs in both diseases. We found that both diseases can cause lymphocyte activation and increase the level of plasma cells. Hence, our study provides genetic evidence to support the possible causal relationship of the two diseases, and periodontitis may increase the host susceptibility and complications to COVID-19. However, further research would be needed to confirm these hypotheses. In this context, we believe that common TFs can provide potential treatment targets, and immunotherapy may provide new treatment insights of the two diseases. Furthermore, preventive oral hygiene measurements and periodontal care could play a role in preventing COVID- 2019 infections and complications.

## Supplementary Information

**Additional file 1:** **Supplementary table 1.** The 1616 differentially expressed genes between periodontitis and healthy control. **Supplementary table 2.** The 10201 differentially expressed genes between COVID-19 and healthy control. **Supplementary table 3.** The 175 pairs of co-upregulated TF between periodontitis and COVID-19.

**Additional file 2:** **Supplementary figure 1.** The immune cell subtypes among periodontitis and healthy control. **Supplementary figure 2.** The immune cell subtypes among COVID-19 and healthy control.

## Data Availability

All data generated or analysed during this study are included in this published article.
