# Supplementary material for: The underlying molecular mechanisms and biomarkers between periodontitis and COVID-19
Source: BMC Oral Health. 2023 Jul 26;23:524. doi: 10.1186/s12903-023-03150-4 (PMC10369766; doi:10.1186/s12903-023-03150-4)

Supplementary figure 1: The immune cell subtypes among periodontitis and healthy control

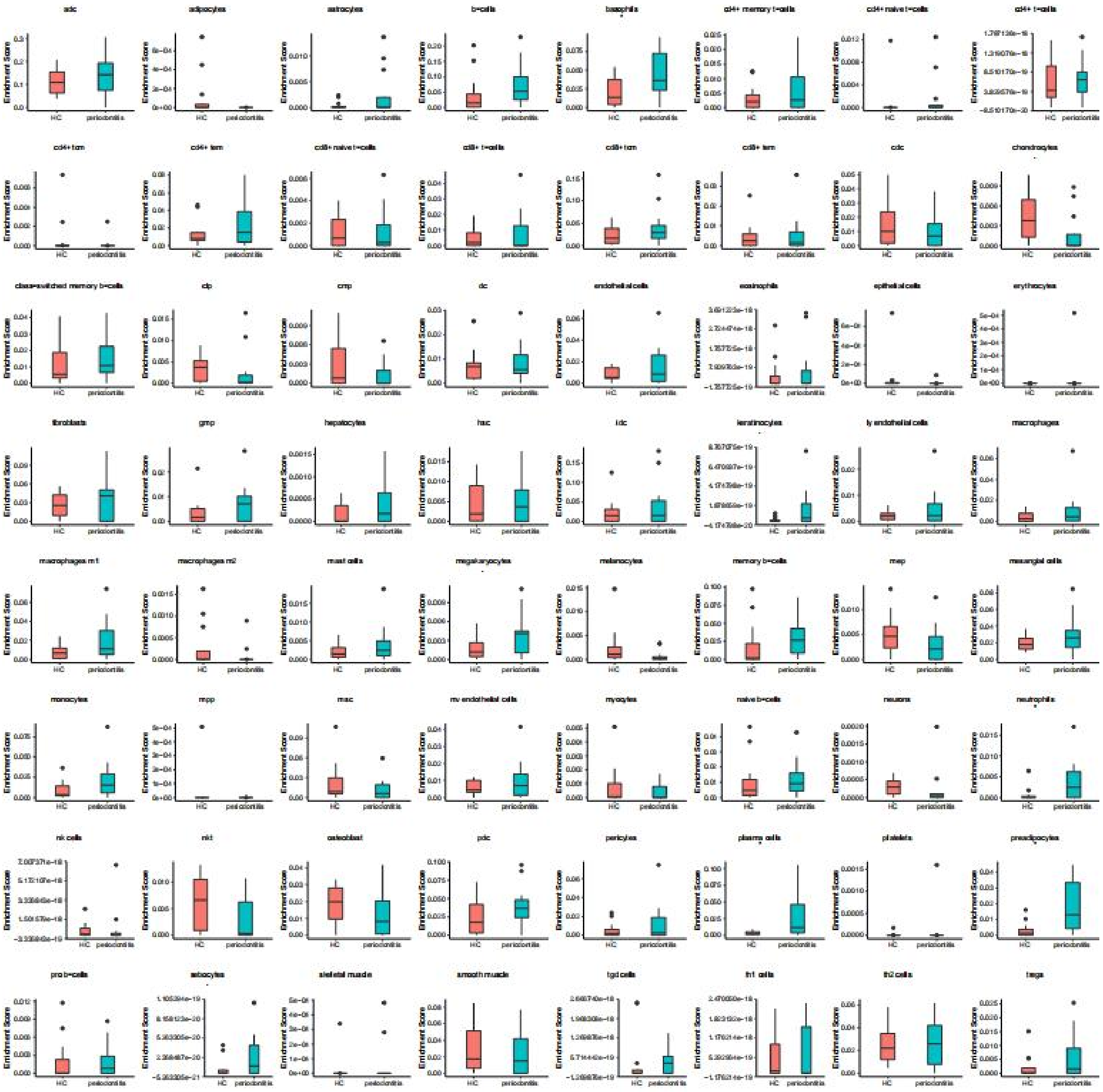

Supplementary figure 2: The immune cell subtypes among COVID-19 and healthy control

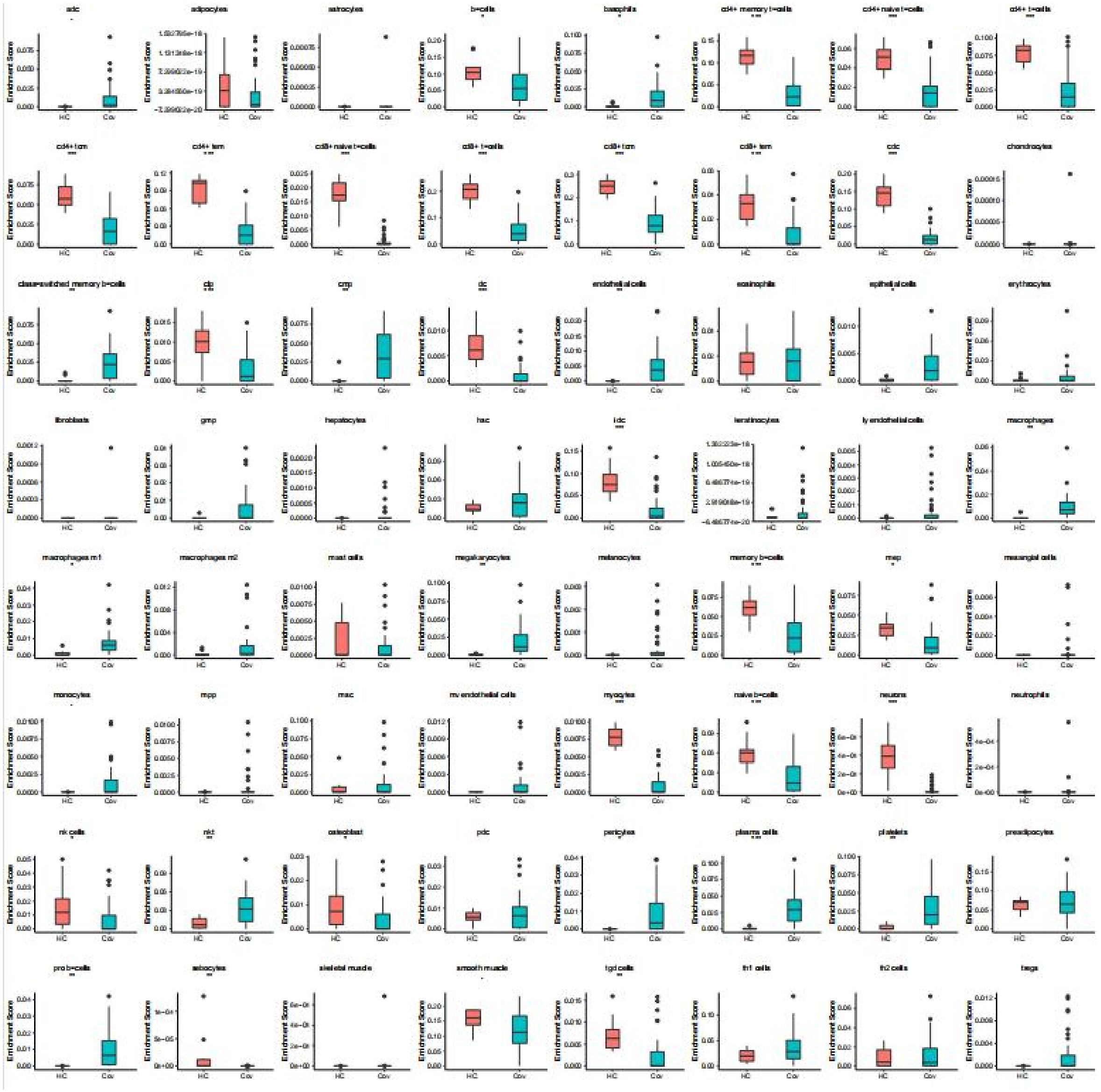

Supplement: Supplementary file 2 — Additional file 2: Supplementary figure 1. The immune cell subtypes among periodontitis and healthy control. Supplementary figure 2. The immune cell subtypes among COVID-19 and healthy control. [file 12903_2023_3150_MOESM2_ESM.pdf]
